# Gender differences in the control of multiple cardiovascular disease risk factors in type 2 diabetes patients

**Published:** 2016

Adjusted mean diastolic blood pressure levels were found to be significantly higher in women compared to men, but other risk factors were almost the same between genders, according to recent research.

Williams and colleagues conducted a cross-sectional study in which patients were randomly recruited from three primary care clinics in the south-eastern USA and asked to complete a self-report survey yielding data relevant to gender differences in cardiovascular disease (CVD) risk-factor control. The primary outcomes were individual diabetes-related risks, which were defined as not having an HbA1c level < 7%, blood pressure of < 130/80 mmHg, and low-density lipoprotein (LDL) cholesterol level < 100 mg/dl (2.59 mmol/l), and composite control defined as having all three outcomes under control simultaneously.

Of the patients enrolled, 56% were men, 67% were non-Hispanic black, and 78% made less than $35 000 per year. Unadjusted mean systolic blood pressure (134 vs 13 mmHg, p = 0.005) and LDL cholesterol levels [99.7 vs 87.6 mg/dl (2.58 vs 2.27 mmol/l), p < 0.001] were much higher in women than in men; however, after adjusting for relevant confounders, differences in systolic blood pressure and LDL cholesterol levels were not significant. Adjusted mean diastolic blood pressure levels were found to be significantly higher in women compared to men (β = 3.09, 95% CI = 0.56–5.63).

Regarding the gender differences in composite control, the results showed that women had poorer control of multiple CVD risk outcomes than men (β = 2.90, 95% CI = 1.37–6.13). Other primary outcomes were not statistically significantly different, including glycaemic control in both genders.

Limitations of this study included the fact that the crosssectional study design did not prove causal associations. Also, confounders not controlled for included diabetes knowledge, self-management practices, medication adherence, co-morbidity burden, social support, duration of diabetes, medications used to treat diabetes, and hypertension. In addition, high triglyceride level was an independent risk factor for coronary heart disease, particularly for women.

In conclusion, further study is needed. In the meantime, both genders, but especially women, need to be encouraged to adopt healthy lifestyle habits with a view to modifying their risk factors and achieving better outcomes.
